# Ki-67 is a valuable prognostic predictor of lymphoma but its utility varies in lymphoma subtypes: evidence from a systematic meta-analysis

**DOI:** 10.1186/1471-2407-14-153

**Published:** 2014-03-05

**Authors:** Xin He, Zhigang Chen, Tao Fu, Xueli Jin, Teng Yu, Yun Liang, Xiaoying Zhao, Liansheng Huang

**Affiliations:** 1 Department of Hematology, the Second Affiliated Hospital, Zhejiang University School of Medicine, Hangzhou, China; 2 Department of Oncology, the Second Affiliated Hospital, Zhejiang University School of Medicine, Hangzhou, China; 3 Department of Oral surgery, the Second Affiliated Hospital, Zhejiang University School of Medicine, Hangzhou, China

**Keywords:** Ki-67, Prognostic value, Lymphoma, Meta-analysis

## Abstract

**Background:**

Ki-67 is a nuclear protein involved in cell proliferation regulation, and its expression has been widely used as an index to evaluate the proliferative activity of lymphoma. However, its prognostic value for lymphoma is still contradictory and inconclusive.

**Methods:**

PubMed and Web of Science databases were searched with identical strategies. The impact of Ki-67 expression on survival with lymphoma and various subtypes of lymphoma was evaluated. The relationship between Ki-67 expression and Diffuse Large B Cell Lymphoma (DLBCL) and Mantle Cell Lymphoma (MCL) was also investigated after the introduction of a CD-20 monoclonal antibody rituximab. Furthermore, we evaluated the association between Ki-67 expression and the clinical-pathological features of lymphoma.

**Results:**

A total of 27 studies met the inclusion criteria, which comprised 3902 patients. Meta-analysis suggested that high Ki-67 expression was negatively associated with disease free survival (DFS) (HR = 1.727, 95% CI: 1.159-2.571) and overall survival (OS) (HR = 1.7, 95% CI: 1.44-2) for lymphoma patients. Subgroup analysis on the different subtypes of lymphoma suggested that the association between high Ki-67 expression and OS in Hodgkin Lymphoma (HR = 1.511, 95% CI: 0.524-4.358) was absent, while high Ki-67 expression was highly associated with worse OS for Non-Hodgkin Lymphoma (HR = 1.777, 95% CI: 1.463-2.159) and its various subtypes, including NK/T lymphoma (HR = 4.766, 95% CI: 1.917-11.849), DLBCL (HR = 1.457, 95% CI: 1.123-1.891) and MCL (HR = 2.48, 95% CI: 1.61-3.81). Furthermore, the pooled HRs for MCL was 1.981 (95% CI: 1.099-3.569) with rituximab and 3.123 (95% CI: 2.049-4.76) without rituximab, while for DLBCL, the combined HRs for DLBCL with and without rituximab was 1.459 (95% CI: 1.084-2.062) and 1.456 (95% CI: 0.951-2.23) respectively. In addition, there was no correlation between high Ki-67 expression and the clinical-pathological features of lymphoma including the LDH level, B symptoms, tumor stage, extranodal site, performance status and IPI score.

**Conclusions:**

This study showed that the prognostic significance of Ki-67 expression varied in different subtypes of lymphoma and in DLBCL and MCL after the introduction of rituximab, which was valuable for clinical decision-making and individual prognostic evaluation.

## Background

Lymphomas represent a highly heterogeneous group of hematological malignancies that can be classified into two major categories: Hodgkin lymphoma (HL) and non-Hodgkin lymphoma (NHL). NHL can be further classified into subgroups such as Diffuse Large B Cell Lymphoma (DLBCL), Follicular Lymphoma (FL), Mantle Cell Lymphoma (MCL), NK/T cell lymphoma and so on [1]. Over the past decades, the incidence of lymphoma has increased dramatically, with NHL becoming the seventh most common form of cancer in the United States [2]. However, although prognostic factors based on clinical-pathological characteristics have been widely used in predicting survival of patients with NHL, including Ann Arbor staging and the international prognostic index (IPI), the precisely survival predictors on the basis of biological markers are still lacking [3]. Therefore, identifying more biomarkers to precisely stratify the group of patients with poorer outcome and thus formulate the individually treatment regimens is necessary and urgent.

Ki-67, a nuclear nonhistone protein, is synthesized at the beginning of cell proliferation, and it is expressed in all phases of the cell cycle except during G0 phase [4]. Its strict association with cell proliferation and its co-expression with other well-known markers of proliferation indicate a pivotal role in cell division. Ki-67 expression has been widely used in clinical practice as an index to evaluate the proliferative activity of lymphoma. However, the relationship between Ki-67 expression and outcome with various subtypes of lymphoma are still contradictory and inconclusive in various studies. Some studies show that high Ki-67 expression correlates with poorer survival rates, while others show no association or the reverse results [5-10]. Moreover, the finding that the predictive significance of some prognostic factors changed following the introduction of a CD-20 monoclonal antibody, rituximab, underscores the necessity for revaluating the prognostic value of predictive factors after the introduction of rituximab [11,12]. Therefore, further investigation is necessary to clearly delineate the relationship between Ki-67 expression and prognosis in lymphoma.

In this study, we performed a meta-analysis to explore the impact of Ki-67 expression on survival with various subtypes of lymphoma including HL, DLBCL, MCL, FL and NK/T cell lymphoma. In addition, the relationships between Ki-67 expression and DLBCL and MCL were investigated after the introduction of rituximab. Furthermore, we also evaluated the association between Ki-67 expression and the clinical-pathological features of lymphoma. The results of our study provide valuable information for the prognosis evaluation and clinical treatment regimen making in lymphoma.

## Methods

### Literature search

PubMed and Web of Science databases were searched with the following terms: “Ki67”, “Ki-67”, “MIB-1”, “lymphoma” and “prognosis”. The most recent search update was 31 August 2013. After examining the titles and abstracts of the relevant articles and excluding nonrelated articles, full-text checking of resting articles was performed. The references of all of the included articles were also evaluated to find additional relevant studies.

### Inclusion and exclusion criteria

Strict inclusion criteria were used in identifying eligible studies. Studies were included if they met the following requirements: (1) The study investigated the association between Ki-67 expression in tumor samples and overall survival (OS), disease free survival (DFS) or clinical-pathological features of the lymphoma; (2) The study provided sufficient data that the hazard ratio (HR) of the OS and the DFS or the odds ratio (OR) of the clinical-pathological factors could be calculated along with the corresponding 95% confidence interval (CI) (3) The study used immunohistochemistry (IHC) as a measurement technique (this criterion was implemented to avoid discrepancies resulting from use of different assay methods to measure Ki67) (4) The study results were written in English.

Only research complying with all of the above inclusion criteria was finally included in our meta-analysis. Thus, reviews, case reports, editorials or letters to the editor without original data were not included, and studies with detection methods such as polymerase chain reaction (PCR) or techniques other than IHC, as well as articles published in a language other than English were excluded.

### Data extraction and assessment of study quality

Two primary investigators (CZG and FT) independently conducted data extraction on the basis of the Preferred Reporting Items for Systematic Reviews and Meta-Analyses (PRISMA) statement [13]. Any discrepancies were resolved by reviewing the study together and reaching a consensus. The following information was retrieved from each study: first author, year, country, age of patients, disease subtype, treatment regimen, number of total cases and number of high Ki-67 expression and low Ki-67 expression patients, high Ki-67 expression threshold, study design, HR (95CI%) of OS or DFS and clinical-pathological data. The quality of each study included in our meta-analysis was assessed according to the Newcastle–Ottawa quality assessment scale [14].

### Statistical analysis

We calculated the pooled HRs and the 95% CI (confidence interval) to analyze the aggregated impact of Ki-67 expression on the survival outcome of lymphoma. Both the DFS and OS were counted. Moreover, subgroup analyses were also conducted based on the various subtypes of lymphoma. HRs and their 95% CI were calculated using the extracted data with the methods described in Parmar’s study [15]. Essentially, if the HR and the corresponding 95% CI were not reported directly, data were extracted from the survival curve published in the article and then estimated using Engauge Digitizer version 4.1 (http://digitizer.sourceforge.net/). We also calculated the ORs and their 95% CI to assess the correlation between Ki-67 expression and the clinical-pathological features of lymphoma, such as performance status (PS), IPI score, stage, B Symptom, LDH level and extranodal site. An observed HR > 1 indicates a worse survival prognosis for the group with high Ki-67 expression, while an observed OR < 1 implies unfavorable clinical features in the high Ki-67 expression group. If the 95% CI not crossing 1, the correlation of Ki-67 expression with survival or clinical-pathological features was considered statistically significant.

The statistical heterogeneity between the trials included in the meta-analysis was assessed by the Chi-square based Q statistical test according to Peto’s method [16]. Inconsistency was quantified using the inconsistency index (I^2^) statistic. A p-value less than 0.10 for the Q-test indicates substantial heterogeneity among the studies. Random-effects or fixed-effects models were used depending on the heterogeneity of the included studies. In the presence of substantial heterogeneity, the pooled ORs and HRs were calculated by the random-effects model (the DerSimonian and Laird method) [17]. Otherwise, the fixed-effects model (the Mantel-Haenszel method) was used [18]. Egger’s test was used to detect possible publication bias and publication bias was supposed to exist when Egger’s test yielded a p value <0.05. All calculations were performed by STATA version 12.0 software (Stata Corporation, Collage Station, Texas, USA). A two-tailed P value of less than 0.05 was considered statistically significant.

## Results

### Literature search and study characteristics

**Figure 1:**
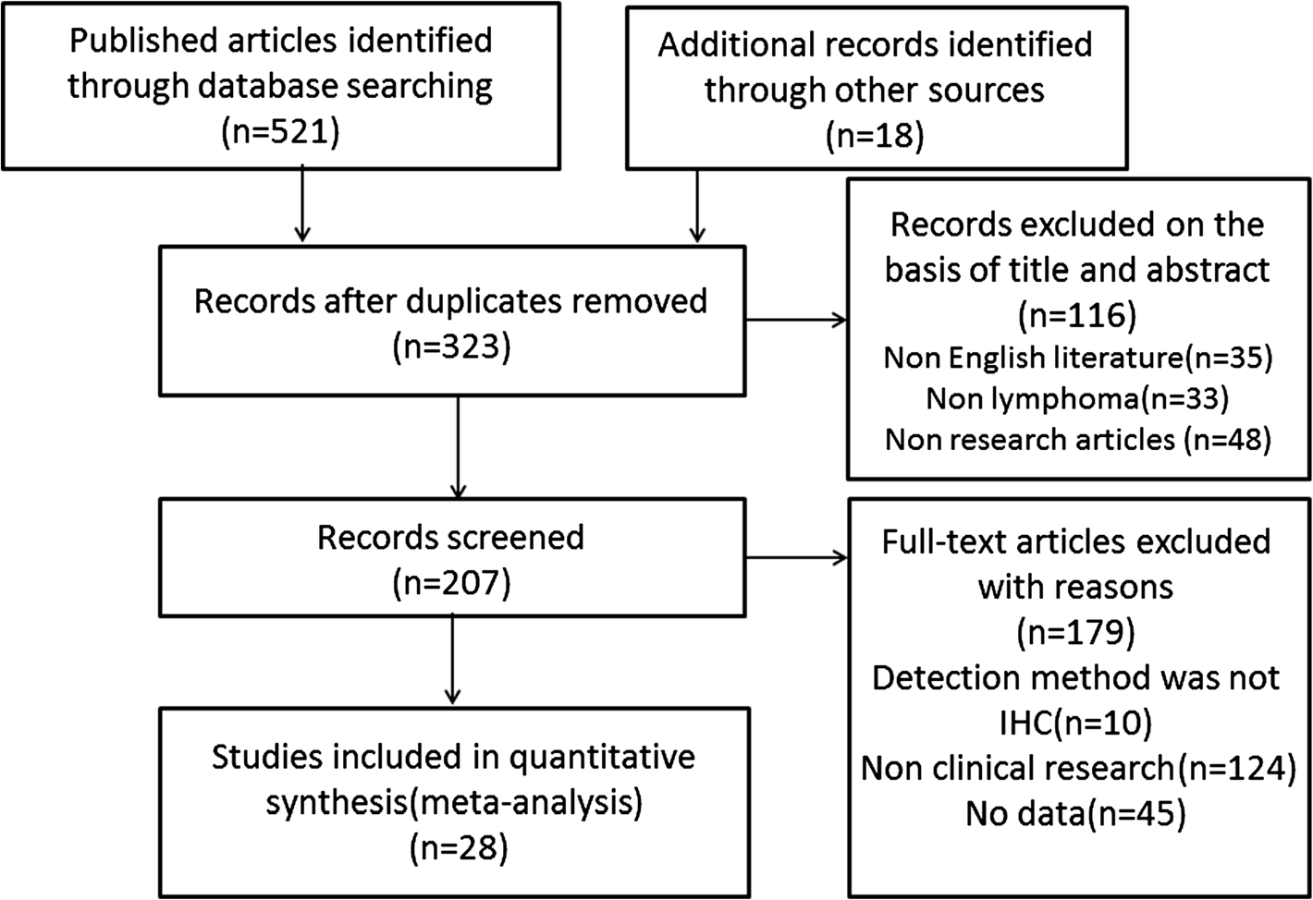
Flow diagram of the relevant studies selection procedure.

We identified 539 potentially relevant articles through the search strategy presented in Figure 1. After screening on the basis of abstracts or titles and then reviewing full-text, 511 studies were excluded. Thus, 28 eligible studies were finally included in this systematic review and meta-analysis [5-10,19-40].

The details of the 28 studies are shown in Table 1. A total of 4112 patients were enrolled in these studies which were published between 1990 and 2013. Of these 28 studies, 5 each were conducted in America, 4 in Spain, 3 in Korea, one each in Italy China, Germany, Canada, Czech, Sweden, Poland, Japan, Egypt and Finland, and the rest 6 studies were conducted in Europe including many countries. 11 studies were prospective, while 17 were retrospective. The subclasses of lymphoma studied are as follows: HL was studied in 3 articles and NHL was in 25 articles. Of the 25 articles investigating NHL, 12 focused on DLBCL, 9 on MCL, 2 on NK/T cell lymphoma, and 2 articles examined various lymphoma subtypes.

**Table 1:** Characteristics of studies included in the final meta-analysis of Ki-67 expression and prognosis of lymphoma

**First author**	**Year**	**Country**	**Patient (P/N)**	**Age (year)**	**Disease subtype**	**High Ki-67 expression threshold**	**Antibody**	**Sample source**	**Therapy regimen**	**HR (95% CI) of OS**	**HR (95% CI) of DFS**	**Quality score**	**Study type**
Salek	2013 [40]	Czech	210 (74/136)	NA	MCL	30% (estimated)	Anti-MIB-1	WTS	From no chemotherapy to intensive induction with or without ASCT	2.05 (1.45-2.89)	NA	7	R
									R-treated	2.63 (1.72-4.04)	NA		
Li	2013 [5]	America	43 (18/25)	46 median	NK/TL	60% (image analysis)	Anti-Ki-67	WTS	CHOP + RT mostly	5.15 (1.52-17.58)	3.43 (1.1-10.72)	6	R
Geisler	2012 [6]	Europe	122 (33/89)	56 median	MCL	33% (estimated)	NA	NA	R-CHOP/R-AraC + ASCT	2.64 (1.46-4.77)	2.32 (1.39-3.89)	7	p
Li	2012 [7]	China	118 (55/63)	53 median	DLBCL	70% (estimated)	Anti-Ki-67	WTS	R-CHOP + RT	1.284 (1.075-1.535)	3.38 (1.64-6.93)	8	R
Salles	2011 [19]	Europea and America	1138 (627/511)	NA	DLBCL	75% (estimated)	NA	TMAs	R-CHOP	1.8 (1.1-2.9)	NA	8	p
									CHOP	1 (0.6-1.5)	NA		
Gaudio	2011 [20]	Italy	111 (38/73)	60 median	DLBCL	80% (estimated)	NA	WTS	R-CHOP	2.1 (1.6-4.8)	2.6 (1.2-3.8)	6	R
									CHOP	2.1 (0.9-4.3)	NA		
Song	2011 [21]	Korea	65 (31/34)	59 median	DLBCL	70% (estimated)	Anti-Ki-67	WTS	HD-MTx + WBRT	1.657 (0.893-3.259)	NA	5	R
Ott	2010 [22]	Europe	386 (283/103)	68 median	DLBCL	75% (estimated)	Anti-MIB-1	TMAs	R-CHOP	0.8 (0.4-1.5)	0.8 (0.5-1.4)	7	P
									CHOP	1.1 (0.6-1.9)	0.9 (0.5-1.4)		
Barros	2010 [23]	Germany	87 (53/34)	14 median	HL	50% (image analysis)	Anti-MIB-1	TMAs	ABVD + RT	NA	NA	7	R
Goy	2010 [8]	Europea and America	67 (16/51)	66 median	MCL	50% (estimated)	Anti-Ki-67	WTS	Bortezomib	1.93 (0.9-4.1)	NA	5	P
Yoon	2010 [24]	Korea	144 (46/98)	54 median	DLBCL	85% (estimated)	Anti-Ki-67	WTS	R-CHOP + partial RT	2.876 (0.972-8.508)	2.9 (1.26-6.7)	9	P
Chung	2010 [25]	Canada	80 (22/58)	64 median	MCL	50% (estimated)	Anti-Ki-67	WTS	CHOP mostly	4.4 (2.24-8.64)	NA	4	R
Garcia	2008	America	76 (38/38)	62 median	MCL	20% (estimated)	Anti-Ki-67	WTS	R-hyper-CVAD	NA	NA	9	P
His	2008	America	52 (17/35)	56 median	MCL	35% (estimated and image analysis)	Anti-Ki-67	WTS	R-CHOP + ASCT	NA	2.46 (1.14-5.31)	9	P
Hasselblom	2008 [10]	Sweden	199 (149/50)	66 median	DLBCL	49% (image analysis)	Anti-Ki-67	WTS	CHOP/CHOP like + partial RT	0.625 (0.256-1.11)	NA	8	P
Szczuraszek	2008 [28]	Poland	53 (24/29)	56.3 mean	NHL	30% (image analysis)	Anti-MIB-1	WTS	NR	2.567 (1.144-5.761)	NA	6	R
Determann	2008 [29]	Europe	249 (nr)	NA	MCL	10% (estimated)	Anti-MIB-1 or Ki-S5	NA	CHOP or MCP or R-CHOP	1.27 (1.15-1.39)	NA	6	P
Kim	2007 [30]	Korea	50 (27/23)	41 median	NK/TL	65% (estimated)	Anti-MIB-1	WTS	CHOP + partial RT	4.33 (1.108-16.918)	3.23 (1.07-9.72)	8	R
Bahnassy	2006 [31]	Egypt	50 (35/15)	45 median	NHL	30% (estimated)	Anti-MIB-1	WTS	CHOP + partial RT	0.696 (0.274-1.766)	NA	7	R
Jerkeman	2004 [9]	Europe	185 (69/116)	NA	DLBCL	60% (estimated)	Anti-MIB-1	WTS	CHOP or M-BACOP	0.56 (0.3-1)	0.53 (0.31-0.91)	5	P
Martinez	2004 [32]	Spain	80 (nr)	NA	MCL	50% (estimated)	Anti-MIB-1	WTS	NA	4.4 (1.9-9.9)	NA	4	R
Seki	2003 [33]	Japan	27 (14/13)	59.7 mean	DLBCL	40% (estimated)	Anti-Ki-67	WTS	CHOP		NA	6	R
Provencio	2003 [34]	Spain	42 (nr)	29mean	HL	20% (NA)	NA	WTS	MOPP or ABVD	3.3 (0.85-12.5)	2.21 (0.92-5.33)	4	R
Raty	2002 [35]	Finland	96 (31/65)	NA	MCL	26% (estimated)	Anti-Ki-67	WTS	CHOP or M-BACOD mostly + partial RT and SCT	3.25 (1.9-5.58)	NA	5	R
Sanchez	1998 [36]	Spain	77 (36/41)	62 median	DLBCL	20% (image analysis)	Anti-MIB-1	WTS	CHOP + partial RT or surgery	1.74 (1.064-2.848)	1.06 (0.48-2.33)	6	R
Morente	1997 [37]	Spain	140 (nr)	NA	HL	Continuous variable (image analysis)	Anti-MIB-1	WTS	MOPP or ABVD + RT	1.04 (1–1.08)	NA	4	R
Miller	1994 [38]	America	60 (11/49)	NA	DLBCL	80% (estimated)	Anti-Ki-67	WTS	CHOP, M-BACOD, ProMACE-CytaBOM, or M-BACOP	5.9 (2.2-16.1)	NA	8	P
Slymen	1990 [39]	America	105 (19/86)	58 median	DLBCL	60% (estimated)	Anti-Ki-67	WTS	CHOP, M-BACOD, ProMACE-CytaBOM, CVP or M-BACOP + partial RT or surgery	3.64 (1.66-7.95)	NA	8	R

P/N, the number of positive/negative; NK/TL. NK/T cell lymphoma; MCL, mantle cell lymphoma; DLBCL, difftuse largeB-cell lymphoma; NHL, Non Hodgkin Lymphoma; HL, Hodgkin Lymphoma; NA, not availiable; WTS, whole tissue sections; TMAs, tissue microarrays; HR, hazard ratio; 95CI%, 95% confidence interval; OS, overall survival; DFS, disease free survival; P, prospective; R, retrospective; R-CHOP, rituximab, cyclophosphamide, doxorubicin, vincristine and prednisone; RT, radiotherapy; ASCT, autologous stem cell transplantation; WBRT, Whole Brain Radiotherapy; ABVD, adriamycin, bleomycin, vinblastine, and dacarbazine; R-hyper-CVAD, rituximab plus hyperfractioned cyclophosphamide, vincristine, doxorubicin, and dexamethasone; MCP, mitoxantrone, chlorambucil, and prednisone; M-BACOD(P), methotrexate, bleomycin, doxorubicin, cyclophospha-mide, vincristine, dexamethasone (prednisone); MOPP, mechlorethamine, vincristine, procarbazine, and prednisone; ProMACE-CytaBOM, doxorubicin, cyclophosphamide, etopo-side, cytarabine, bleomycin, vincristine, methotrexate with leucovorin rescue, and prednisone.

Of the 28 eligible studies, 24 provided the HR of the OS and 11 provided the HR of the DFS directly or indirectly. Three studies investigated the relationship between Ki-67 expression and the survival from DLBCL in two distinct groups, one group with rituximab and one without. Thus, the HR of OS or DFS could be calculated separately for each group and information could be obtained regarding how rituximab influences the relationship between Ki67 expression and prognosis.

### Methodological quality of the studies

The quality of the 28 studies, including case–control and cohort studies, was evaluated based on the Newcastle–Ottawa scale (NOS). The NOS scoring system consists of three components: selection, comparability, and exposure or outcome. The quality scores for selected studies ranged from 4 to 9, and the median score was 6. Fourteen studies (eight prospective and six retrospective) were ranked ‘high quality’, meaning that they scored higher than a six (Table 1).

### Impact of high Ki-67 expression on the survival of lymphoma and the subgroup and the sensitivity analyses

**Figure 2:**
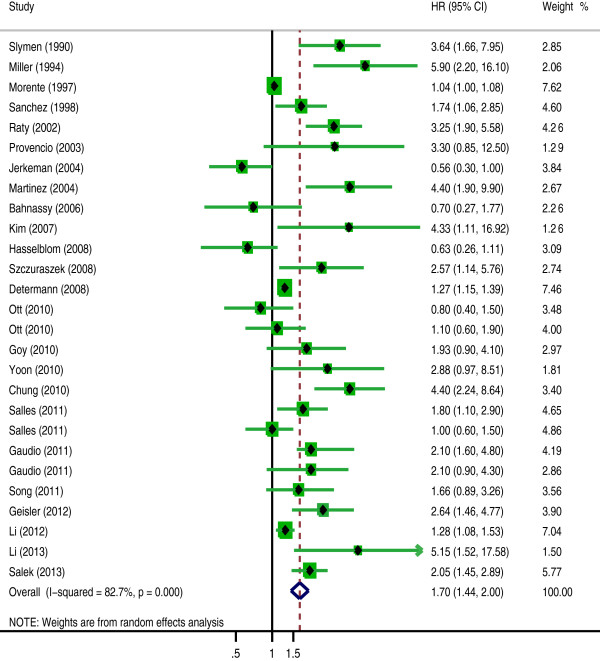
**The hazard ratio (HR) of Ki-67 expression associated with the overall survival (OS).** HR > 1 implied worse OS for the group with high Ki-67 expression.

**Figure 3:**
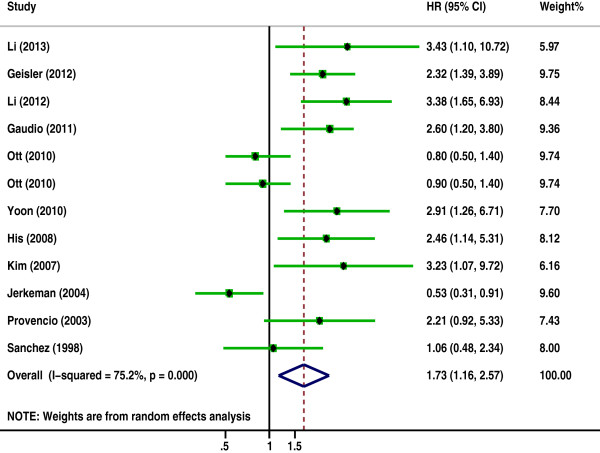
**The hazard ratio (HR) of Ki-67 expression associated with the disease free survival (DFS).** HR > 1 indicated worse DFS for the group with high Ki-67 expression.

The pooled HRs of the OS provided in 24 articles was 1.7 (95% CI: 1.44-2, p = 0.000) (Figure 2) with heterogeneity (I^2^ 82.7% P = 0.000), and the combined HRs of the DFS in 11 articles was 1.727 (95% CI: 1.159-2.571, p = 0.007) (Figure 3) with heterogeneity (I^2^ 75.2% P = 0.000). In both cases, the results demonstrate that high Ki-67 expression is negatively associated with the survival prognosis of lymphoma.

Subgroup analysis of OS was performed according to publication era, study location, number of patients, positive threshold, quality score and study design (Table 2). The results revealed that the negative association between high Ki-67 expression and OS was present across all strata.

**Table 2:** Stratified analysis of the pooled hazard ratio (HR) for the associations of high Ki-67 expression with overall survival (OS) of lymphoma

					**Heterogeneity**	
**Stratified analysis**	**Number of studies**	**Number of patients**	**Pooled HR(95% CI)**	**P value**	**I**^**2**^**(%)**	**P value**
Publication era						
Published in 21^st^	20	3488	1.689(1.386-2.057)	0.000	72.7%	0.000
Published in 20^th^	4	382	1.671(1.41-1.98)	0.000	88,2%	0.000
Study location						
Europe and America	19	3443	1.749(1.447-2.114)	0.000	84.9%	0.000
Asia	4	377	1.694(1.089-2.635)	0.019	44.1%	0.147
Number of patients						
≥100	12	2394	1.35(1.144-1.593)	0.00	80.8%	0.000
<100	12	1476	2.663(1.923-3.687)	0.000	49.3%	0.027
High expression threshold						
≥50%	14	2332	1.906(1.429-2.543)	0.000	73.4%	0.000
<50%	9	1098	1.722(1.238-2.395)	0.001	76.2%	0.000
Quality score						
≥7	11	2582	1.584(1.188-2.112)	0.002	70.9%	0.000
<7	13	988	1.815(1.451-2.271)	0.000	85.9%	0.000
Study design						
P	9	2550	1.336(1.001-1.782)	0.049	70.8%	0.000
R	15	1320	2.144(1.628-2.824)	0.000	85.8%	0.000

The outcome of sensitivity analysis of OS showed that the gathered HR ranged from 1.67 (95% CI: 1.4 – 1.99) to 1.87 (95% CI: 1.49 – 2.35) after removing the study of Chung et al. and the study of Determann et al. respectively (Table 3). It suggested that the negative association between high Ki-67 expression and prognosis of lymphoma existed after excluding anyone study.

**Table 3:** Summary estimates of the hazard ratio (HR) and 95% CI of sensitivity analysis of overall survival (OS) for the meta-analysis

**Study omitted**	**Estimated HR**	**Low value of 95% CI**	**High value of 95% CI**
Slymen (1990) [39]	1.6952128	1.4201434	2.023561
Miller (1994) [38]	1.6858797	1.4149985	2.0086172
Morente (1997) [37]	1.8667241	1.5154643	2.2994003
Sanchez (1998) [36]	1.7437564	1.4534081	2.092108
Raty (2002) [35]	1.6774691	1.4068271	2.0001767
Provencio (2003) [34]	1.7260282	1.4445214	2.0623949
Jerkeman (2004) [9]	1.8312747	1.52944	2.1926765
Martinez (2004) [32]	1.6864121	1.414325	2.010843
Bahnassy (2006) [31]	1.7862245	1.4921515	2.1382532
Kim (2007) [30]	1.7185497	1.439151	2.052191
Hasselblom (2008) [10]	1.8081217	1.5098257	2.1653519
Szczuraszek (2008) [28]	1.7200329	1.438033	2.0573335
Determann (2008) [29]	1.8672591	1.4851215	2.3477252
Ott (2010) [22]	1.8099216	1.5055193	2.1758716
Goy (2010) [8]	1.7372652	1.4506446	2.0805168
Yoon (2010) [24]	1.7240045	1.4422258	2.0608363
Chung (2010) [25]	1.6677585	1.4008845	1.9854728
Salles (2011) [19]	1.7680753	1.4731554	2.1220372
Gaudio (2011) [20]	1.7206854	1.4363353	2.0613279
Song (2011) [21]	1.7479872	1.4580686	2.0955524
Geisler (2012) [6]	1.7052457	1.4261276	2.0389924
Li (2012) [7]	1.8065342	1.4892573	2.1914051
Li (2013) [5]	1.7072812	1.430741	2.0372725
Salek (2013) [40]	1.7172997	1.4338397	2.0567975

### Correlation of high Ki-67 expression with the OS of the subtypes of lymphoma

Of the 24 articles investigating the association between Ki-67 expression and OS, 2 examined Hodgkin Lymphoma and 23 examined Non-Hodgkin Lymphoma. The overall HRs for Hodgkin Lymphoma was 1.511 (95% CI: 0.524-4.358, p = 0.445), with heterogeneity (I^2^ 64.7% P = 0.092), and for Non-Hodgkin Lymphoma, it was 1.777 (95% CI: 1.463-2.159, p = 0.000), with heterogeneity (I^2^ 74.3% P = 0.000) (Table 4). A subsequent subtype analysis of NHL was performed. The pooled HRs for DLBCL, MCL and NK/TL were 1.457 (95% CI: 1.123-1.891, p = 0.005), 2.48 (95% CI: 1.61-3.81, p = 0.000) and 4.766 (95% CI: 1.917-11.849, p = 0.001) respectively (Table 4).

**Table 4:** Summary estimates of the hazard ratio (HR) for the associations of high Ki-67 expression with overall survival (OS) of subtypes of lymphoma

					**Heterogeneity**	
**Subtypes of lymphoma**	**Number of studies**	**Number of patients**	**Pooled HR(95% CI)**	**P value**	**I**^**2**^**(%)**	**P value**
HL	2	162	1.511(0.524-4.358)	0.445	64.7%	0.092
NHL	22	3708	1.777(1.463-2.159)	0.000	74.3%	0.000
DLBCL (total)	11	2588	1.457(1.123-1.891)	0.005	67.7%	0.000
DLBCL (with rituximab)	5	753	1.495(1.084-2.062)	0.014	52.3%	0.078
DLBCL (without rituximab)	9	1835	1.456(0.951-2.23)	0,084	74.8%	0.000
MCL (total)	7	904	2.48(1.61-3.81)	0.000	85.6%	0.000
MCL (with rituximab)	3	581	1.981(1.099-3.569)	0.023	87.4%	0.000
MCL (without rituximab)	3	243	3.123(2.049-4.76)	0.000	21.8%	0.278
NK/TL	2	93	4.766(1.917-11.849)	0.001	0%	0.853

### Correlation of high Ki-67 expression with the introduction of rituximab

**Figure 4:**
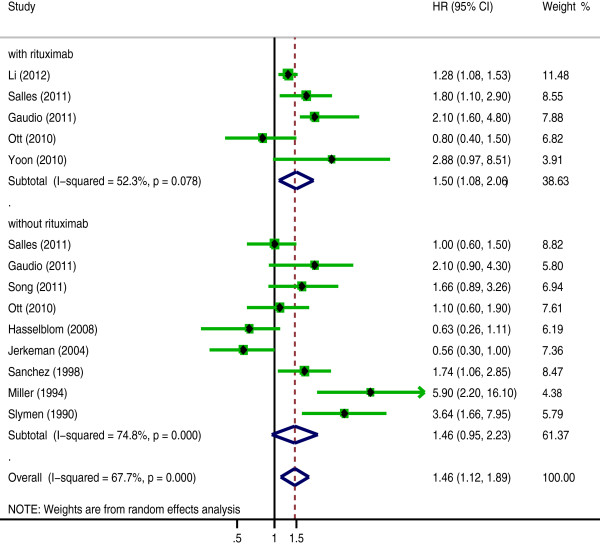
**The hazard ratio (HR) of Ki-67 expression associated with the overall survival (OS) of DLBCL following and without following rituximab treatment.** HR > 1 implied worse OS for the group with high Ki-67 expression.

**Figure 5:**
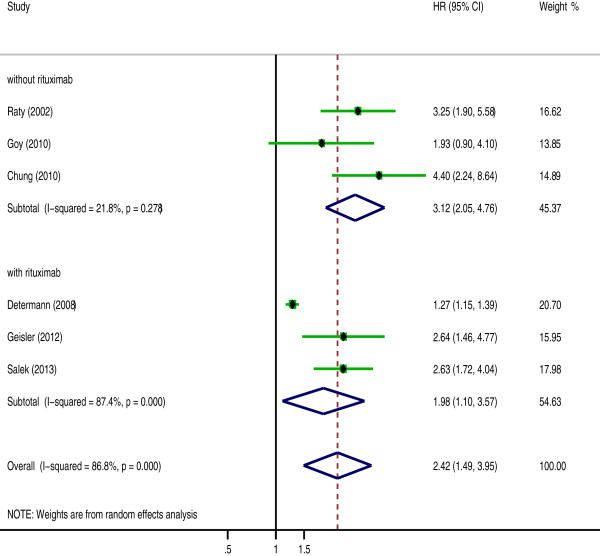
**The hazard ratio (HR) of Ki-67 expression associated with the overall survival (OS) of MCL following and without following rituximab treatment.** HR > 1 implied worse OS for the group with high Ki-67 expression.

The effect of rituximab treatment on the association between the Ki-67 expression and the OS was also evaluated for DLBCL and MCL. The combined HRs for DLBCL with rituximab was 1.459 (95% CI: 1.084-2.062, p = 0.014), compared to 1.456 (95% CI: 0.951-2.23, p = 0.084) for DLBCL without rituximab (Figure 4, Table 4). In contrast, with MCL, the prognostic significance of Ki-67 expression was not changed after the introduction of rituximab; the pooled HRs was 1.981 (95% CI: 1.099-3.569, p = 0.023) with rituximab and 3.123 (95% CI: 2.049-4.76, p = 0.000) without rituximab (Figure 5, Table 4).

### Association between high Ki-67 expression and clinical-pathological characteristics of lymphoma

Overall, there was no correlation between high Ki-67 expression and the clinical-pathological features of lymphoma. Ten studies investigated the association of high Ki-67 expression with the LDH level and B symptoms. The pooled ORs were 0.914 (95% CI: 0.673-1.241, P = 0.564) and 0.851 (95% CI: 0.557-1.3, P = 0.455) for LDH level and B symptoms respectively (Table 5). To analyze the correlation of Ki-67 expression with tumor stage and the extranodal site, nine studies were used. The aggregated ORs were 1.089 (95% CI: 0.79-1.501, P = 0.604) for tumor stage and 1.017 (95% CI: 0.674-1.534, P = 0.937) for extranodal site (Table 5). Finally, seven studies evaluated the correlation of high Ki-67 expression with performance status and IPI score. The combined ORs were 1.082 (95% CI: 0.654-1.793, P = 0.758) for performance status and 0.963 (95% CI: 0.667-1.39, P = 0.84) for IPI score, revealing no correlation for either clinical-pathological feature (Table 3).

**Table 5:** Summary estimates of the odds ratio (OR) for the associations of High Ki-67 expression and clinical-pathological features of lymphoma

					**Heterogeneity**	
**Clinicopathological features**	**Nuber of studies**	**Nuber of patients**	**Pooled OR(95% CI)**	**P value**	**I**^**2**^**(%)**	**P value**
Performance status (0–1 vs.2-5)	7	643	1.082(0.654-1.793)	0.758	31.7	0.186
Elevated LDH (normal vs. elevated)	10	884	0.914(0.673-1.241)	0.564	36.6	0.115
IPI score (0–2 vs.3-5)	7	611	0.963(0.667-1.39)	0.84	0	0.53
Tumor stage (I-II vs. III-IV)	9	845	1.089(0.79-1.501)	0.604	37.4	0.119
B symptom (no vs. yes)	10	944	0.851(0.557-1.3)	0.455	45	0.059
Extranodal site (no vs. yes)	9	758	1.017(0.674-1.534)	0.937	20.7	0.259

### Publication bias

**Figure 6:**
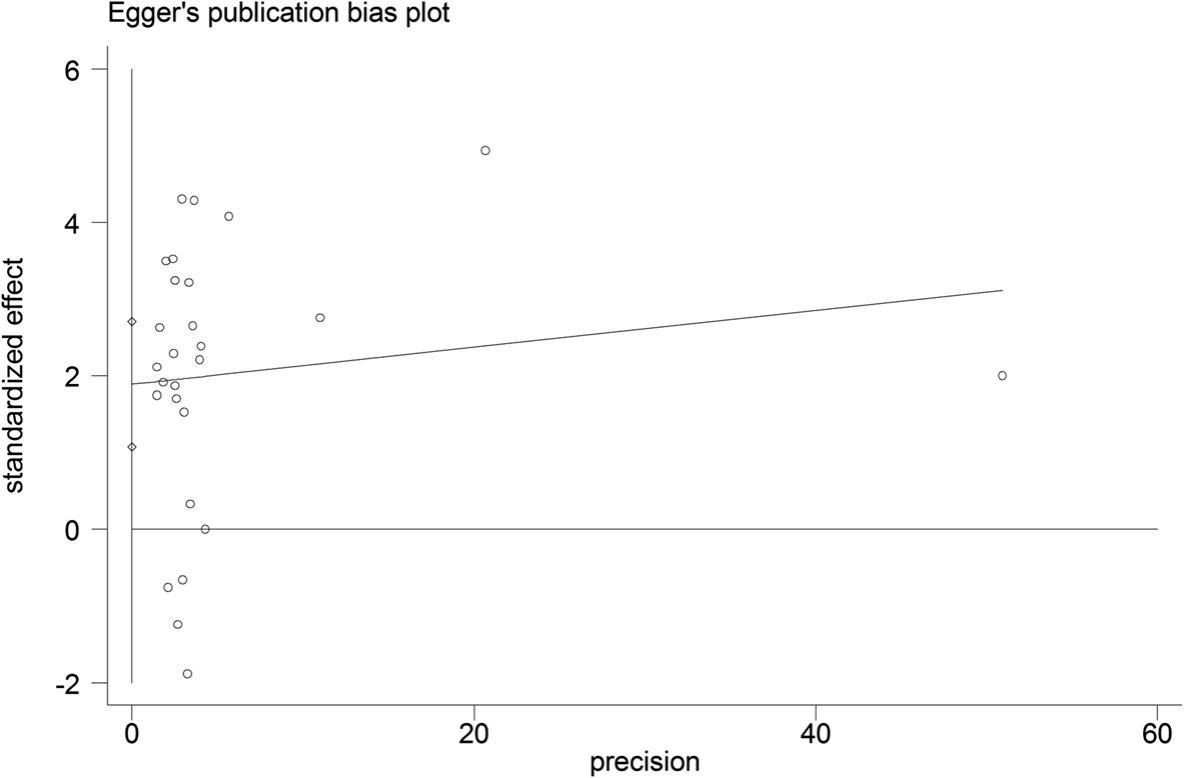
Funnel plot of Ki-67 expression and the overall survival (OS) of lymphoma for the assessment of publication bias.

Egger’s test showed no publication bias for high Ki-67 expression with regard to OS, DFS and the clinical-pathological features of lymphoma. Figure 6 provides a funnel plot for the study on the effects of high Ki-67 expression on the OS of lymphoma.

## Discussion

Ki-67 is a nuclear nonhistone protein first identified in 1991 by Gerdes et al. [41]. Because it is expressed in all phases of the cell cycle except the resting stage (G0), it has been used as a proliferation marker in numerous cancers including lymphoma [38,42,43]. Nonetheless, studies examining the relationship between Ki-67 expression and prognosis with lymphoma have been inconclusive. In this study, we conducted a meta-analysis that pooled data from all of the relevant studies to explore the correlation between Ki-67 expression and the survival outcome in lymphoma. The results revealed that high Ki-67 expression in patients with lymphoma was associated with worse prognosis, both for the OS (HR = 1.7, 95% CI: 1.44-2; P = 0.000) and DFS (HR = 1.727, 95%CI: 1.159-2.571; P = 0.007). Sensitivity analysis suggested that the association between high Ki-67 expression and prognosis of lymphoma was stable and not changed after removing anyone study. Additionally, when subgroup analysis was performed according to study design, the prospective studies which exhibited more powerful statistics still showed a statistically significant prognostic value for Ki-67 expression in lymphoma (HR = 1.336, 95% CI: 1.001-1.782; P = 0.049).

Lymphoma is a type of hematological malignancy displaying substantial heterogeneity, and the clinical and biological characteristics of the different subtypes vary greatly. In our meta-analysis, the outcome of further analyzing the association between Ki-67 expression and the prognosis of various subtypes of lymphoma supported it. High Ki-67 expression was a valuable prognostic indicator for NHL (HR = 1.777, 95% CI: 1.463-2.159, P = 0.000) and its various subtypes such as DLBCL, MCL and NK/TL, but not for HL (HR = 1.511, 95% CI: 0.524-4.358, P = 0.445).

The development of new therapeutic agents, such as rituximab, that target specific molecules, has greatly improved the survival rates with lymphoma. According to the cancer statistics provided by American Cancer Society in 2012, the 5-year survival rate of NHL has improved from 47% in 1970s to 70% in recent years, and rituximab has been central to this improved prognosis.[2]. However, it is important to realize that after treatment with rituximab, many widely used and validated prognostic factors no longer remain significant in lymphoma [11,12]. Ann Arbor stage III-IV, age, B symptoms and serum LDH level, which predict survival in chemotherapy groups not taking rituximab, are no longer accurate predictors in chemotherapy groups taking rituximab. In our study, owing to the limited data, we only analyzed the effect of rituximab on the prognostic significance of Ki-67 expression in DLBCL and MCL. In DLBCL, without rituximab, Ki-67 expression was not related to OS rates, consistent with the results of the Nordic Lymphoma Group Study and other additional studies [9,20-22]. However, the prognostic value of Ki-67 expression in DLBCL became significant following rituximab treatment (HR = 1.459, 95% CI: 1.084-2.062, p = 0.014). A recent large prospective study from the Lunenburg Lymphoma Biomarker Consortium including 2451 patients also identified high Ki-67 expression as a good predictive factor in DLBCL with rituximab, which is consistent with our findings [19]. In MCL, the difference was observed: high Ki-67 index was related to a poor OS with rituximab (HR = 1.981, 95% CI: 1.099-3.569, p = 0.023), and was also correlated with survival outcome without following rituximab treatment (HR = 3.123, 95% CI: 2.049-4.76, p = 0.000).

Though our study yielded important results regarding the prognostic value of Ki-67 expression in lymphoma, some limitations existed in our meta-analysis. First, although subgroup analysis adjusted for the cut-off point was conducted and no changing of the prognostic value of Ki-67 was found, the heterogeneity caused by the different cut-off point of high Ki-67 expression was inevitably. Therefore, the high inter-observer variability still restricts the use of the Ki-67 index in experimental as well as clinical practice relatively. Spyratos et al. suggested to choose the cut-off point according to the clinical objective [44]. That is: to exclude patients with slowly proliferating tumors, low cut-off point should be set to avoid overtreatment, while high cut-off point is suitable to be used to identify patients sensitive to chemotherapy schedule. However, an optimal cut-off point needs to be defined and validated for lymphoma further. Second, we were unable to evaluate the relationship between Ki-67 expression and the treatment outcome due to the limited data. This is important, as some authors speculate that the association between high Ki-67 expression and poor prognosis in lymphoma results from regrowth of tumors or an increased likelihood of future mutations, leading to treatment failure, whereas others suggest that high proliferative activity, represented by high Ki-67 expression, may be more sensitive to chemotherapy and thus more likely to respond well to treatment[9,30,45]. Therefore, it would be interesting to investigate the correlation between high Ki-67 expression and the treatment outcome to clarify the mechanism of Ki-67 action. In addition, another limitation was the inability to evaluate certain subtypes of lymphoma due to the paucity of studies related to them.

## Conclusion

Despite these limitations, this meta-analysis supports the prognostic value of Ki-67 in lymphoma, demonstrating a significant correlation between high Ki-67 expression and a poor survival outcome. However, this association exists in the NHL subtypes, including DLBCL, MCL, NK/TL, but not in HL. In addition, in DLBCL, Ki-67 expression has prognostic value following rituximab treatment but the prognostic value diminishes without following rituximab treatment, while in MCL, the prognostic value of Ki-67 expression exists whether or not following rituximab treatment. Thus, the prognostic significance of Ki-67 expression varies in different subtypes of lymphoma and in DLBCL and MCL after the introduction of rituximab, which is valuable for individual prognostic evaluation. Further adequately designed prospective studies still need to be conducted to verify our results.

## Competing interests

The authors declare that they have no conflict of interest.

## Authors’ contributions

XH CZ and LH contributed to study design and data preparation. XH, TF and XJ performed data extraction and data analysis. XH, TF, XZ and XJ were involved in manuscript preparation, paper writing and the manuscript revision. All authors read and approved the final manuscript.

## Pre-publication history

The pre-publication history for this paper can be accessed here:

http://www.biomedcentral.com/1471-2407/14/153/prepub
